# Circulatory arrest time above 30 min have significantly detrimental effects on the outcomes of type a aortic dissection repair

**DOI:** 10.1186/s13019-025-03819-7

**Published:** 2026-02-17

**Authors:** Abhishek Chakraborty, Aparna Ragupathi, Sorasicha Nithikasem, Damion Anglin, Seung Woo Baek, Hirohisa Ikegami, Gengo Sunagawa, Ashok Chaudhary, Mark Russo, Leonard Y. Lee, Anthony Lemaire

**Affiliations:** Department of Surgery, Division of Cardiothoracic Surgery, Rutgers Robert Wood Johnson Medical School, 125 Paterson Street, New Brunswick, NJ USA

## Abstract

**Background:**

Acute aortic dissection of the ascending aorta is a life-threatening disease that poses a significant challenge for cardiovascular surgeons. Dissection of the aorta typically occurs when the aortic media separates from the intima. Surgical repair is performed emergently and classically involves the use of hypothermic circulatory arrest for distal aortic repair. The impact of circulatory arrest duration on postoperative outcomes is unclear with the critical time leading to increased risk being controversial. The purpose of this study is to elucidate the pivotal circulatory arrest time that increases surgical complications in patients undergoing type A aortic dissection repair.

**Methods:**

This retrospective review of prospectively collected data included patients who underwent Aortic Dissection Repair from 2016 to 2022 at a New Jersey institution. Circulatory arrest time groups were stratified by above and below 30 min. Primary outcomes included 30-day mortality, postoperative length of stay (LOS), 30-day readmission and 12-month mortality. Secondary outcomes included postoperative complications of acute kidney injury (AKI), pericardial or pleural effusion, postoperative cerebrovascular accident (CVA) and postoperative atrial fibrillation. Outcomes were analyzed using Pearson’s Chi-squared, Fisher’s Exact, Regression Analysis and Pooled T-Tests, with significance set at *p* < 0.05.

**Results:**

A total of 109 patients were included, 87 of whom (80%) had arrest times below 30 min and 22 (20%) had arrest times above 30 min. There were no differences in preoperative baseline characteristics besides in patients with a history of congestive heart failure (*p* = 0.015). There were differences in cardiopulmonary bypass time (*p* < 0.001) and cross clamp time (*p* < 0.001). Patients with circulatory arrest times less than 30 min had a lower rate of 30-day mortality (*p* < 0.01), 12-month mortality (*p* < 0.019) and CVA (*p* = 0.003). There was no effect of cerebral perfusion strategy, retrograde vs. anterograde vs. lack thereof, during circulatory arrest on rate of CVA (*p* = 0.982).

**Conclusions:**

Circulatory arrest time above 30 min increases the risk of postoperative mortality and CVA. Further investigation into evaluating these patients long-term should be pursued in addition to developing strategies to minimize circulatory arrest times to under 30 min.

## Introduction

Type A aortic dissection is a rare but highly morbid condition characterized by the separation of the layers of the aortic wall, specifically the tunica intima from tunica media forming an intimal flap otherwise known as a false lumen. Blood flow into this aberrant anatomic space then propagates the dissection and induces hemodynamic instability leading to systemic malperfusion [1, 2]. Emergent open surgical repair is the first-line intervention for the management of Stanford type A aortic dissections involving the ascending aorta and arch. A variety of surgical techniques are employed, usually involving prosthetic vascular graft interposition with possible coronary reimplantation and aortic valve repair depending on the degree of aortic root involvement [3].

As first described by DeBakey et al., for a type A aortic dissection repair, the surgeon must first establish cardiopulmonary bypass (CPB) before repairing the tear by transecting the area of dissection, obliterating the false lumen and completing anastomosis of the aorta [4]. This sequence of maneuvers can prove challenging for surgeons who must work to balance the veracity of repair technique with the likelihood postoperative complications in the setting of an emergent surgery with limited time for preoperative planning. Factors such as length of dissected intimal flap and involvement of root, coronary ostia, or great vessels can increase the repair complexity [5, 6]. Many of the postoperative complications associated with type A aortic dissection repairs including arrhythmias, acute kidney injury (AKI), as well as pericardial and pleural effusions overlap with those of patients undergoing other cardiac surgeries [2, 5].

Cerebrovascular accident (CVA), in particular, is a uniquely common complication in type A aortic dissection repairs compared to other cardiac surgery procedures. Incidence of CVA in type A aortic dissection repairs, has been shown to be as high as 30% in some studies [7]. The pathogenesis of CVA is attributable to a period of complete corporeal circulatory arrest, during which flow through the cardiopulmonary bypass circuit is paused while the heart is arrested, in order to facilitate the distal anastomosis of the prosthetic aortic graft [8]. CPB is then reinitiated for the proximal anastomosis to limit cerebral and visceral ischemia time. While DeBakey et al. pioneered the initial aortic surgeries with circulatory arrest, it was found that cerebral tissue was particularly sensitive to the imposed ischemia resulting in high rates of postoperative neurological dysfunction. Griepp et al. later modified Debakey et al.’s method to include induced deep hypothermia, with corporeal cooling to as low as 15° C, during circulatory arrest (DHCA) for the purpose of neuroprotection [9]. By inducing systemic deep hypothermia, tissue metabolic demand is significantly reduced thereby limiting cerebral and visceral tissue damage during DHCA. More recently, studies have concluded that the protective mechanism of deep hypothermia alone does not adequately protect patients from postoperative neurologic dysfunction. Thus, special neurologic protection strategies performed concomitantly with deep hypothermia have been developed such as selective cerebral perfusion and the administration of pharmaceutical neuroprotectants [10–13]. The safe duration of DHCA even with these new strategies, however, remains unclear with the threshold of DHCA leading to untenable risk being controversial [7,8,14–17]. Additionally, most studies investigating circulatory arrest duration during aortic surgery focus on elective aortic repairs for which outcomes cannot be extrapolated to the more morbid population of type A aortic dissection patients requiring emergent surgery. In this retrospective review, we investigate the impact of DHCA time on risk of postoperative complications for patients undergoing emergent type A aortic dissection repair.

## Methods

This retrospective review included all patients with type A aortic dissection who underwent emergent repair from January 2016 to May 2022. This study was performed at a single high-volume academic institution designated as a tertiary care center for all cardiac pathologies. Outcomes were assessed for patients with DHCA less than 30 min, and for patients with DHCA greater than 30 min. The modes of cerebral protection include (1) Antegrade cerebral protection through (a) an axillary artery cannulation, and (b) direct cannulation of the innominate artery with balloon-tipped perfusion catheters. The second mode of cerebral protection is Retrograde cerebral perfusion through the superior vena cava. The Retrograde cerebral perfusion goal was for 150–300 ml/min and a central venous pressure (CVP) measurement of 25-30mmHg.

Primary outcomes included 30-day mortality, postoperative length of stay (LOS), 30-day readmission and 12-month mortality. Secondary outcomes included postoperative complications of AKI, pericardial or pleural effusion, postoperative CVA and postoperative atrial fibrillation. Perioperative characteristics were also included in this analysis to assess their association and contribution to postoperative outcomes. The characteristics included were CPB time, circulatory arrest time, cross-clamp time, Debakey Type I vs. II subclassification, deep hypothermic temperature and cerebral perfusion strategy. Intragroup and comparative analysis was performed. Statistical analysis was conducted using Pearson’s Chi Square test, Fisher’s Exact test, Student’s T-Test and Linear regression with significance set at p levels of < 0.05.

All of the surgeries were performed by a total of 5 surgeons during the time period. Each surgery involved hemi-arch replacement of the aorta and no patients had total aortic arch replacement. During the circulatory arrest period, all patients received steriods, and ice was placed on the patient’s head. Cerebral oximetry was used on all patients.

## Results

Our study included 109 patients presenting with type A aortic dissections who underwent emergent surgical repair. Of those 109 patients, 87 (80%) patients underwent a DHCA time of under 30 min during the repair while 22 (20%) patients underwent a DHCA time of greater than 30 min. Our analysis evaluated baseline characteristics (Table 1) at initial presentation, which consisted of age at time of surgery, sex, race, and body mass index (BMI). There were no statistical differences noted with regard to any baseline characteristics. The preoperative comorbidities (Table 1) evaluated were hypertension, hyperlipidemia, diabetes mellitus type I/II, history of CVA, congestive heart failure, smoking and diagnosis of COPD. Congestive heart failure was the only preoperative comorbidity in which we found a difference between the circulatory arrest time groups (6% (*n* = 5) vs. 23% (*n* = 5), (*p* = 0.015)).

**Table 1:** Baseline characteristics

Variable		Overall (*n* = 109)	Circulatory Arrest Less than 30 min (*n* = 87)	Circulatory Arrest Greater than 30 min (*n* = 22)	*P* -Value
Baseline characteristics					
	Age (years) (Median, IQR)	63 (53–72)	62 (53–78)	66 (53–80)	0.109^
	Sex (male) n (%)	109	73	36	0.70
	Race (White) n %	58 (42%)	48 (82%)	10 (17%)	0.764^^
	Race (African American) n %	19 (14%)	14 (74%)	5 (26%)	0.764^^
	Race (Asian) n %	12 (9%)	11 (92%)	1 (8%)	0.764^^
	Race (Hispanic) n %	14 (10%)	12 (86%)	2 (14%)	0.764^^
	Race (Other)	36 (26%)	29 (81%)	7 (19%)	0.764^^
	Body Mass Index (Median, IQR)	29 (25–32)	29	27	0.418^
Comorbidities					
	Hypertension n (%)	82 (75%)	64 (74%)	18 (82%)	0.423
	Hyperlipidemia n (%)	21 (27%)	17 (28%)	4 (22%)	0.608
	Diabetes Mellitus Type I/II n (%)	27 (25%)	24 (28%)	3 (14%)	0.176
	Preoperative Atrial Fibrillation n (%)	27 (25%)	22 (26%)	5 (23%)	0.761
	Previous Cerebrovascular Accident n (%)	11 (10%)	7 (8%)	4 (18%)	0.178
	History of Congestive Heart Failure n (%)	10 (9%)	5 (6%)	5 (23%)	**0.015**
	History of Smoking n (%)	32 (30%)	28 (33%)	4 (18%)	0.178
	Diagnosis of COPD n (%)	8 (7%)	7 (8%)	1 (5%)	0.567

*Indicates significance at *p* < 0.05, No ^ indicates Pearson’s Chi Square Test, ^ indicates Student’s T-Test, ^^indicates Fisher’s Exact

The variables included in our analysis of perioperative characteristics (Table 2) included CPB in minutes, circulatory arrest time in minutes, subclassification of type A dissections as Debakey Type I versus Debakey Type II, deep hypothermia temperature, and circulatory arrest selective cerebral perfusion delivered in antegrade versus retrograde fashion versus lack thereof. Our analysis demonstrated significant differences in CPB Time (Median: 135 vs. 212 min (*p* < 0.001), circulatory arrest time (Median: 20 vs. 44 min (*p* < 0.001), cross clamp time (Median: 86 vs. 123 min (*p* = 0.015)), and hypothermic temperature (Median: 25° C vs. 25° C (*p* = 0.036). There was no difference in Debakey Type I versus II classification (54 vs. 15 (*p* = 0.595)). All patients who were not classified as Debakey Type I were classified as Debakey Type II. Our analysis found no significant differences in delivery of antegrade versus retrograde versus no cerebral perfusion during DHCA (*p* = 0.982).

**Table 2:** Perioperative characteristics

Variable		Overall (*n* = 109)	Circulatory arrest less than 30 min (*n* = 87)	Circulatory arrest greater than 30 min (*n* = 22)	*P* -value
Perioperative characteristics					
	Cardiopulmonary Bypass Time (minutes) (Median, IQR)	143 (115–208)	135 (113–184)	212 (174–255)	< 0.001*^
	Circulatory Arrest Time (minutes) (Median, IQR)	22 (18–27)	20 (17–23)	44 (36–55)	< 0.001*^
	Cross-clamp Time (minutes) (Median, IQR)	92 (70–133)	86 (67–120)	123 (98–172)	**0.015^**
	Debakey Type I	69 (63%)	54 (62%)	15 (68%)	**0.595**
	Debakey Type II	40 (37%)	33 (38%)	7 (32%)	**0.595**
	Deep Hypothermia Temperature (Median, IQR)	25 (24–26)	25 (24–26)	25 (22–26)	0.036*^
	Anterograde Circulatory Arrest Cerebral Perfusion n (%)	77 (72%)	61 (71%)	16 (73%)	0.982
	Retrograde Circulatory Arrest Cerebral Perfusion n (%)	16 (15%)	13 (15%)	3 (14%)	0.982
	No Circulatory Arrest Cerebral Perfusion n (%)	15 (14%)	12 (14%)	3 (14%)	0.982

*Indicates significance at *p* < 0.05, No ^ indicates Pearson’s Chi Square Test, ^ indicates Student’s T-Test

The primary outcomes (Table 3) of 30-Day Mortality, postoperative length of stay, 30 Day readmission and 12-month postoperative mortality were also analyzed. Amongst the primary outcomes measured, only 30-day mortality (14% (*n* = 12) vs. 41% (*n* = 9) (*p* < 0.001) and 12-month mortality (12% (*n* = 10) vs. 32% (*n* = 7) (*p* < 0.001) were significantly different between the two groups. Finally, our analysis evaluated the secondary outcomes (Table 3) of AKI, postoperative pericardial or pleural effusion, postoperative CVA and postoperative atrial fibrillation. We found a statistically significant difference in the rate of postoperative CVA (8% (*n* = 7) vs. 32%(*n* = 7), (*p* = 0.003)). There were no statistically significant differences in AKI (*p* = 0.834), postoperative effusion (*p* = 0.340) or postoperative atrial fibrillation (*p* = 0.299).

**Table 3:** Postoperative outcomes

Variable		Overall (*n* = 109)	Circulatory arrest less than 30 min (*n* = 87)	Circulatory arrest greater than 30 min (*n* = 22)	*P* -value
Outcomes					
	30-Day Mortality n (%)	21 (19%)	12 (14%)	9 (41%)	< 0.001*
	Postoperative Length of Stay (Days) (Median, IQR)	9	7 (4–13)	6 (1–13)	0.552^
	30- Day Readmission n (%)	21 (20%)	17 (20%)	4 (19%)	0.959
	12 Month Postoperative Mortality n (%)	17 (16%)	10 (12%)	7 (32%)	0.019*
Postoperative complications					
	Acute Kidney Injury n (%)	23 (21%)	18 (21%)	5 (23%)	0.834
	Postoperative Pericardial/Pleural Effusion n (%)	44 (41%)	37 (43%)	7 (32%)	0.340
	Postoperative Cerebrovascular Accident n (%)	14 (13%)	7 (8%)	7 (32%)	**0.003**
	Postoperative Atrial Fibrillation n (%)	30 (28%)	22 (25%)	8 (36%)	0.299

*Indicates significance at *p* < 0.05, No ^ indicates Pearson’s Chi Square Test, ^ indicates Student’s T-Test

## Discussion

The study evaluated the postoperative outcomes of DHCA greater than 30 min compared to DHCA lasting less than 30 min. While several studies have investigated the ideal method for cerebral protection during type A aortic dissection repair, the impact of prolonged DHCA duration on outcomes such as postoperative neurologic status remains controversial [7,8,15–17]. While safe duration of DHCA is thought to range from 30 to 60 min, there lacks a clear consensus on the critical threshold for safe DHCA, even with concomitant cerebral perfusion. Our results suggest that DHCA times exceeding 30 min confer an inordinate risk of CVA and mortality.

There were no statistical differences in the baseline demographics, including age, sex, race, and BMI, of our study groups, limiting confounding in our analysis. Both groups also had similar prevalence of comorbidities, with the exception of a statistically significant difference in preoperative diagnosis of heart failure (*p* < 0.015). Additionally, the similar distribution between groups with regard to DeBakey Classification (*p* = 0.595) and cerebral perfusion method (*p* = 0.892) support that neither the complexity of aortic repair nor cerebral perfusion method significantly contributed to the differences in postoperative outcomes. Most importantly, we evaluated for differences in outcomes for patients with DHCA times greater than and less than 30 min. While many studies have proposed a critical threshold of risk, there lacks a consensus. We propose a more constricted timeframe of 30 min of maximal safe DHCA duration in patients who are undergoing hemiarch replacement. Amongst the primary outcomes in Tables 3 and 30-days and 12-month mortality were found to be significantly higher in the group of greater than 30 min. The rate of CVA was also found to be significantly higher in the greater than 30-minute group, suggesting that a safe duration of DHCA is less than 30 min.

One potential mechanism for increased mortality rates observed in our greater than 30 min cohort is organ malperfusion. DHCA is commonly employed to reduce metabolic demand including in cerebral tissue for which metabolism decreases by 6–7% for every 1° C decrease in temperature [10]. Though a protective technique employed to facilitate graft anastomosis, DHCA is still metabolically stressful, inducing ischemia to essential organs such as the brain, kidney and intestines [17, 18]. Despite the use of selective cerebral perfusion becoming more popular, serious complications from ischemia to other nonperfused organs may still seriously affect the long-term prognosis of patients which is likely a driving factor in the high mortality rates observed in our greater than 30 min cohort. Furthermore, although we suspect the complexity of the aortic injury may not play a major role in the outcomes, it is possible that this can have an impact on how the patient recovers.

O’Hara et al. described a similar trend, with each additional 10-minute increment of DHCA conferring significantly increased risk of stroke or death. While the length of DHCA is predicated on the nature of the necessary repair, an understanding of physiologic limitations due to critical DHCA duration should also inform operative decision making for repair strategy. For example, the decision to prophylactically relocate the left carotid and subclavian arteries to a brachiocephalic trunk graft, termed debranching, may extend the duration of DHCA but is not necessarily required to achieve a successful repair [19]. Similarly, electing to pursue operative techniques such as an open distal aortic graft anastomosis with DHCA versus on CPB with cross clamping of the aorta without DHCA, should consider the consequences of prolonged DHCA. Geirsson et al. found that an open-distal anastomosis was superior in terms of short and mid-term survival but carried an increased burden of postoperative complications, likely due to the technique’s necessity of DHCA [20]. Limiting DHCA to less than 30 min retains the long-term benefits of innovative operative techniques while reducing short term postoperative complications. New techniques such as the branched stented anastomosis frozen elephant trunk (B-SAFER) repair have recognized reducing DHCA times as a pivotal component to their successful outcomes, however wide adoption has not yet taken place. In Roselli e*t al’s* analysis of their novel B-SAFER technique, all groups had median DHCA times above 40 min, despite noting the importance of expedience [21]. As type A aortic dissection repair techniques continue to evolve, limiting DHCA to under 30 min should be a central component of operative planning.

## Conclusion

Our results demonstrate that DHCA durations exceeding 30 min result in a markedly higher rate of CVA and death. This finding should inform operative decision making for future patients undergoing type A aortic dissection repair. As repair techniques continue to evolve, the ability to limit DHCA durations to under 30 min should be a major consideration in all future innovations. Further, intraoperative reconstructive strategies to optimize future operations, such as debranching and frozen elephant trunk, should be considered in the context of the high risk of CVA and death when DHCA times exceed 30 min. While prolonged DHCA durations are understandably associated with inferior postoperative outcomes, the determinate duration is unclear with considerable variability. Future studies are necessary to further characterize the optimal safe DHCA duration and further develop an optimal strategy for neuroprotection in surgeries requiring extended DHCA due to repair complexity.

### Limitations

Limitations to this study include its retrospective nature and relative emergence of required surgery. Type A aortic dissections are often heterogeneous in presentation and thus, it is difficult to control all confounding variables in one analysis. Additionally, intraoperative decision making is difficult to assess despite its significant contributions to preoperative, intraoperative and postoperative outcome variables.

## Data Availability

No datasets were generated or analysed during the current study.
